# Effect of a smartphone-based online electronic logbook to evaluate the clinical skills of nurse anesthesia students in Iran: a randomized controlled study

**DOI:** 10.3352/jeehp.2023.20.10

**Published:** 2023-03-31

**Authors:** Ali Khalafi, Nahid Jamshidi, Nasrin Khajeali, Saeed Ghanbari

**Affiliations:** 1 Department of Anesthesiology, School of Allied Medical Sciences, Ahvaz Jundishapur University of Medical Sciences, Ahvaz, Iran; 2 Educational Development Center, Ahvaz Jundishapur University of Medical Sciences, Ahvaz, Iran; 3 Department of Biostatistics and Epidemiology, School of Health, Ahvaz Jundishapur University of Medical Sciences, Ahvaz, Iran; Hallym University, Korea

**Keywords:** Clinical competence, Iran, Nurse anesthetists, Smartphone, Students

## Abstract

**Purpose:**

This study was conducted to evaluate a smartphone-based online electronic logbook used to assess the clinical skills of nurse anesthesia students in Iran.

**Methods:**

This randomized controlled study was conducted after tool development at Ahvaz Jundishapur University of Medical Sciences in Ahvaz, Iran from January 2022 to December 2022. The online electronic logbook involved in this study was an Android-compatible application used to evaluate the clinical skills of nurse anesthesia students. In the implementation phase, the online electronic logbook was piloted for 3 months in anesthesia training in comparison with a paper logbook. For this purpose, 49 second- and third-year anesthesia nursing students selected using the census method were assigned to intervention (online electronic logbook) and control (paper logbook) groups. The online electronic logbook and paper logbook were compared in terms of student satisfaction and learning outcomes.

**Results:**

A total of 39 students participated in the study. The mean satisfaction score of the intervention group was significantly higher than that of the control group (P=0.027). The mean score of learning outcomes was also significantly higher for the intervention than the control group (P=0.028).

**Conclusion:**

Smartphone technology can provide a platform for improving the evaluation of the clinical skills of nursing anesthesia students, leading to increased satisfaction and improved learning outcomes.

## Graphical abstract

[Fig f2-jeehp-20-10]

## Introduction

### Background/rationale

Formative assessment of the clinical performance of nurse anesthesia students during training courses is an indispensable component of their education. This assessment promotes the quick diagnosis of weaknesses and strengths, correction of performance, and improvement of clinical competence [1]. Therefore, valid methods and tools must be used for this purpose so that both the student and the educational program can benefit from such assessment methods [2]. A variety of methods exist for clinical evaluation, the most effective of which is the logbook [3]. Using a logbook, students can record their professional and clinical experiences in accordance with educational goals, facilitating student learning and performance monitoring [4]. A logbook allows the evaluation not only of student learning but also of the program and the quality of clinical education [3]. Although paper logbooks have long been widely used in medical education, they have been rendered impractical since they lack the necessary flexibility for modern training [5]. Limitations of paper logbooks include the loss of logbooks by students, the inconvenience of carrying logbooks during internships, the impossibility of recording and completing procedures on the same day, and the lack of timely feedback from the instructor [3]. Additionally, evaluating and analyzing the large volume of information in paper logbooks is a long and tedious process [6]. Therefore, solutions must be considered to overcome these limitations [3].

Today, the use of electronic logbooks is rapidly increasing [7] because they simplify data analysis and access [8]. In addition, an electronic logbook can serve as a massive archive of information stored in a tiny space [9]. By virtue of their fast performance, online electronic logbooks can not only improve the interaction between instructor and student, but also improve the quality of anesthesia education and facilitate evaluation [5]. Most electronic logbooks have thus far been designed for a computer or a website, and despite the advantages of such logbooks, users cannot always quickly access them [10]. Recently, the use of smartphone technology among new evaluation methods has won increasing popularity in clinical and educational settings [3], and applications installed on these phones have been demonstrated to be portable, fast, and accurate tools for recording and maintaining information [11]. Since a nurse anesthesia student spends his or her internship at multiple centers and must record performance in a logbook at different times and places, the logbook used must be portable and adaptable so that information can be recorded quickly and timely feedback can be given to the student. Such features are made possible by smartphone technology.

### Objectives

The present study was conducted to design, implement, and evaluate a smartphone-based online electronic logbook (the AGAH app) to evaluate the clinical skills of nurse anesthesia students. We hypothesized that by providing quick access, online evaluation, and timely feedback, the design and implementation of the AGAH app would increase the nurse anesthesia students’ satisfaction and improve their learning outcomes.

## Methods

### Ethics statement

This study was approved by the Ethics Committee of Ahvaz Jundishapur University of Medical Sciences (AJUMS) (Ref. ID: IR.AJUMS.REC.1401.280). The objectives, stages, and conditions of the study were fully explained to all participants, and an informed consent form was obtained from each of them.

### Study design

The present study was a randomized controlled study conducted after tool development. It was described according to CONSORT (Consolidated Standards of Reporting Trials) Statement available at https://www.consort-statement.org/.

### Settings

This study began in January 2022 and ended in December 2022. The design and development of the AGAH app took about 8 months. Data were collected for 3 months (from October to December 2022) in the operating rooms of 5 university hospitals affiliated with AJUMS.

### Interventions

The 3 stages of this study were design, implementation, and evaluation. Each of these steps is detailed below.

#### Design phase

The process of designing the AGAH app is shown in Supplement 1.

#### Implementation phase

This study was piloted in the operating rooms of 5 university hospitals affiliated with AJUMS from October 2022 to December 2022 (1 academic semester). At the beginning of the semester, second- and third-year nurse anesthesia students were randomly assigned to intervention (AGAH app) and control (paper logbook) groups. Then, the intervention and control groups were matched in terms of their demographic variables. Afterward, in a 2-hour meeting, both groups were separately taught to use their logbooks. Members of the intervention group installed the application on their smartphones, and participants in the control group received their paper logbooks. In another meeting, the clinical instructors were briefed on evaluating and providing feedback, and the supervising professors and department head were familiarized with their supervisory role. The AGAH app was completely consistent with the paper logbook in content and scoring instructions. Over 3 months, the participants in the intervention and control groups documented their skills in the application and the paper logbook, respectively. As soon as the academic semester ended, the logbooks of the control group were collected, and the user accounts of the intervention group members were deactivated in the AGAH app.

#### Evaluation phase

The intervention and control groups were evaluated and compared based on the variables of satisfaction and learning outcomes. After the intervention, a post-test satisfaction questionnaire was completed by the members of both groups. The learning outcomes of both groups were measured based on the total score of the procedures recorded in the application and in the paper logbook.

### Participants

All 49 second- and third-year anesthesia nursing students of AJUMS, including 14 men (28.5%) and 35 women (71.5%) with a mean age of 21±3.65 years, entered the study. Sampling was done using the census method, and informed consent was obtained from the participants. Participants were excluded from the study if they failed to record their activities in the application or the logbook, lost the paper logbook, or did not participate in any of the research stages. The data of participants who met the exclusion criteria were not included in the final analysis.

### Outcomes

In this study, the following outcomes were investigated: (1) demographic characteristics, (2) the intervention and control groups’ level of satisfaction with the AGAH app and the paper logbook, respectively, and (3) the learning outcomes of the groups after completing the internship.

### Data sources and measurement

To measure the level of satisfaction of the students, a researcher-made satisfaction questionnaire was used. The first section of this questionnaire dealt with demographic information (age, sex, academic year, and overall grade point average). The second section consisted of 20 items scored based on a 5-point Likert scale (from completely disagree [1 point] to completely agree [5 point]), with a minimum score of 20 and a maximum score of 100. The items examined in this questionnaire included the guide for using the logbook, appearance characteristics, ease of use, speed of evaluation and provision of feedback, impact on motivation, impact on self-confidence, educational communication between the student and the clinical instructor, and speed of correction and improvement of clinical performance. Based on this questionnaire, a score between 20 and 39 represented complete dissatisfaction, 40 to 59 relative dissatisfaction, 60 to 79 relative satisfaction, and 80 to 100 complete satisfaction. After the questionnaire was developed based on textbooks, articles, and other reliable sources, its content validity was confirmed by the faculty members of the AJUMS Department of Anesthesiology and Medical Education using a qualitative approach. Then, the questionnaire was piloted to 30 nursing anesthesia students who were not among the final participants. Their comments were applied to confirm the face validity of the tool. Next, the reliability of the questionnaire was confirmed by an obtained Cronbach α coefficient of 0.96 (Supplement 2).

The third section of the tool was an anesthesia skills evaluation checklist used in the logbooks, developed and approved by faculty members based on valid anesthesia sources and a literature review (Supplements 3). This checklist had been used for 5 years to evaluate the skills of AJUMS nurse anesthesia students in anesthesia internships, and the necessary revisions and amendments to the checklist had already been made. In this checklist, the student performance level in each specific skill is scored on a 4-point scale: requires repetition (1 point), average (2 points), good (3 points), and excellent (4 points). The total scores attained by the student (out of 100 points) were regarded as the score of learning outcomes during the internship period. Based on this checklist, a score of 0 to 20 was considered poor, 21 to 40 average, 41 to 60 good, 61 to 80 very good, and 81 to 100 excellent. As mentioned earlier, the AGAH app was also fully consistent with the paper logbook in terms of content and scoring instructions.

### Bias

None.

### Study size

Sample size calculation was performed using G*Power ver. 3.0.10 (University of Düsseldorf), using the independent-samples Student t-test, 2-tailed alpha of 0.05, power (1-β) of 0.80, and effect size (Cohen d) of 0.8. The result indicated that a sample size of approximately 25 participants per group was required. Therefore, all second- and third-year nurse anesthesia students of AJUMS (n=49) were included in the study using the census method. The study included 25 students in the intervention group and 24 students in the control group.

### Randomization

Forty-nine students were randomly assigned to the intervention and control groups. Each student was randomly assigned a code. Then, the codes were placed in a box. The first code drawn from the box conferred assignment to the intervention group, while the second code represented allocation to the control group. This process continued until all students had been selected, at which time 25 students were in the intervention group and 24 in the control group. Subsequently, the intervention and control groups were matched in terms of their demographic variables. The unit of analysis was the same as the unit of assignment (intervention or control).

### Blinding (masking)

No blinding was done.

### Statistical methods

IBM SPSS ver. 25.0 (IBM Corp.) was used for data analysis. The normality of data distribution was confirmed using the Shapiro-Wilk test. Data were analyzed using descriptive (mean, standard deviation, percentage, and frequency) and analytical (independent t-test and chi-square test) statistics. P-values less than 0.05 were considered to indicate statistical significance.

## Results

### Participants

**Fig. 1.**
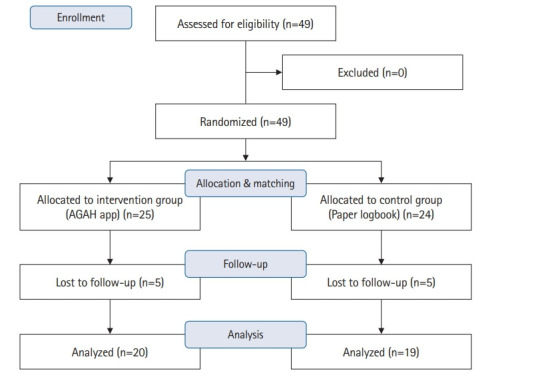
Flowchart of the study.

During the 3 months of the study, of the 49 nursing anesthesia students initially participating, only 39 (20 from the intervention group and 19 from the control group) completed all steps of the study (Fig. 1, Dataset 1). Based on the chi-square test, the intervention and control groups displayed no statistically significant difference in terms of sex (P=0.55, χ^2^=0.34) or academic year (P=0.42, χ^2^=0.64). Furthermore, based on the independent t-test results, no statistically significant difference was present between the groups in terms of age (P=0.13, t=1.53) or grade point average (P=0.72, t=−0.35). The personal and academic characteristics of the participants are detailed in Table 1.

### Main results

The independent t-test was used to compare the mean scores for satisfaction and learning outcomes of the intervention and control groups. The results showed a statistically significant difference between the groups in the mean satisfaction score (Dataset 1), with a significantly higher score in the intervention group than in the control group (P=0.027). A statistically significant difference was also observed between groups in the mean score of learning outcomes (Dataset 2), with the score of the intervention group significantly higher than that of the control group (P=0.028) (Table 2).

## Discussion

### Key results

The aim of the present study was to design, implement, and evaluate a smartphone-based online electronic logbook (the AGAH app) used to evaluate the clinical skills of nurse anesthesia students in Iran and to compare it with a paper logbook. The results of this study indicated that the mean scores for satisfaction and learning outcomes of the intervention group were significantly higher than those of the control group.

### Interpretation

The present study provides a solid answer to the question of how new and technology-based evaluation methods can constitute a suitable alternative to previous evaluation methods in clinical settings. The results showed that the use of a smartphone-based online electronic logbook could lead to higher satisfaction of nurse anesthesia students relative to a paper logbook. The higher satisfaction with this new method can be explained by the quick access of students to sections on their previous activities, versatility of the online electronic logbook for use across different times and places, high accuracy, user-friendliness, and improved interaction and relationship between the student and the clinical instructor or the department head. In general, new and student-centered methods have been reported to be associated with higher student satisfaction in clinical and educational settings [3,12].

Various studies have also pointed out numerous advantages of electronic logbooks, aligning with the results of the present study. Among the main advantages of electronic logbooks are the facilitated analysis of recorded data and the easy archiving of information while occupying minimal physical space. This type of logbook also represents an environmentally friendly green technology, which reduces paper consumption. In contrast, paper logbooks are difficult to transport, occupy considerable space, and can make it difficult to analyze a large amount of information [3,6,9].

Another finding of the present study was improved performance of the nurse anesthesia students in clinical settings due to the use of the smartphone-based online electronic logbook. In the present study, the purpose of designing an online electronic logbook in particular was to enable quick evaluation of student activity, timely feedback to the student, and constant instructor-student interaction. The findings confirmed our hypothesis that the design of a logbook with the described characteristics could positively impact the clinical performance of nurse anesthesia students. Overall, the importance of formative evaluation and timely feedback is indisputable, as it provides students with sufficient opportunities to improve their performance; this is enabled by online electronic logbooks [4]. Accordingly, the improved performance of the nurse anesthesia students in the present study likely originates from the prompt feedback made possible by the online logbook. Therefore, due to their advantages, electronic logbooks constitute a very useful clinical evaluation method and can greatly contribute to the anesthesia profession by promoting the acquisition of skills and facilitating clinical monitoring and evaluation.

### Comparison with previous studies

The findings of the present research provide support for previous studies on electronic logbooks. For instance, a study conducted by Tamblyn et al. [10] in Australia showed that relative to a paper logbook, a smartphone-based electronic logbook improved the compliance of junior doctors in recording information in the logbook. Also, Barbieri et al. [5] examined the compliance of anesthesiology residents in recording clinical activities in a web-based online electronic logbook. They reported that the online electronic logbook can be a useful tool for recording and evaluating the clinical activities of anesthesiology residents. In contrast, in a study conducted in the United States, Straker and Metz [13] investigated the effectiveness of a web-based online electronic logbook to improve airway management training for anesthesiology residents. According to their analysis, the procedures recorded in the advanced airway management training rotation helped the department head make carefully informed decisions to improve training in airway management. The results showed that an electronic logbook can help improve educational programs by enabling the collection of detailed information. Aphinives [6] also conducted a study aimed at designing and implementing a web-based electronic logbook to monitor the training of general surgery assistants in Thailand. The researchers concluded that the use of an electronic logbook led to more efficient access to data, improved performance monitoring of trainers and trainees, and more careful analysis of information regarding procedures performed at different centers [6]. In addition, a recent study in Iran involved a smartphone-based online electronic logbook designed for master’s students of intensive care nursing. Based on the results, the evaluation of clinical performance using smartphone technology was associated with increased student satisfaction [3].

The above studies resemble the current research in the demonstrated effectiveness of converting paper logbooks into electronic ones. However, the research population of the previous studies included postgraduate students or residents in various medical specialties, while the target group in the present study was undergraduate students. In addition, the electronic logbooks used in prior studies were mainly web-based, and very few had been developed as smartphone applications. Also, those studies did not utilize or investigate important benefits of online electronic logbooks, such as quick and timely feedback to students. Several of the abovementioned studies involved only analysis of the information in the electronic logbook and reporting of various statistics, and some addressed only the desire and satisfaction of the students. As Straker and Metz [13] pointed out as a limitation of their study, they did not address the learning outcomes or the clinical competence of the students. Therefore, our study can be argued to be the first to investigate the outcome of student learning via the design and implementation of a smartphone-based online electronic logbook.

### Limitations

Despite its strengths, the present study had a few limitations. First, the low internet speed and frequent disconnections may have affected the results. Additionally, the AGAH app was not compatible with the iOS operating system. Other limitations include the small sample size and the time limit for conducting the study.

### Generalizability

The findings of the present study not only can help optimize the clinical evaluation of nurse anesthesia students, but also may be useful for students of other fields of medical sciences in Iran. The design of the AGAH app allows department heads and professors of other disciplines to define the relevant procedures and activities according to the nature of their field and make desired changes through the AGAH app management panel.

### Suggestions

The present study can be used as a guide to devise cost-effective and up-to-date clinical evaluation methods. Future studies are recommended to investigate the impact of online logbooks (such as the AGAH app) on other variables related to clinical evaluation or among students of other fields of medical sciences. Longitudinal studies with larger sample sizes are expected to yield more definitive results.

### Conclusion

The findings of this study indicate that smartphone technology can be used as a platform for improving the evaluation of nurse anesthesia students’ clinical skills, leading to increased satisfaction and improved learning outcomes. Through the design and development of a smartphone-based online electronic logbook (the AGAH app), the present study enabled online clinical evaluation. This resulted in improved interaction and communication between the student, the clinical instructor, the supervising professor, and the department head.

## Figures and Tables

**Figure f2-jeehp-20-10:**
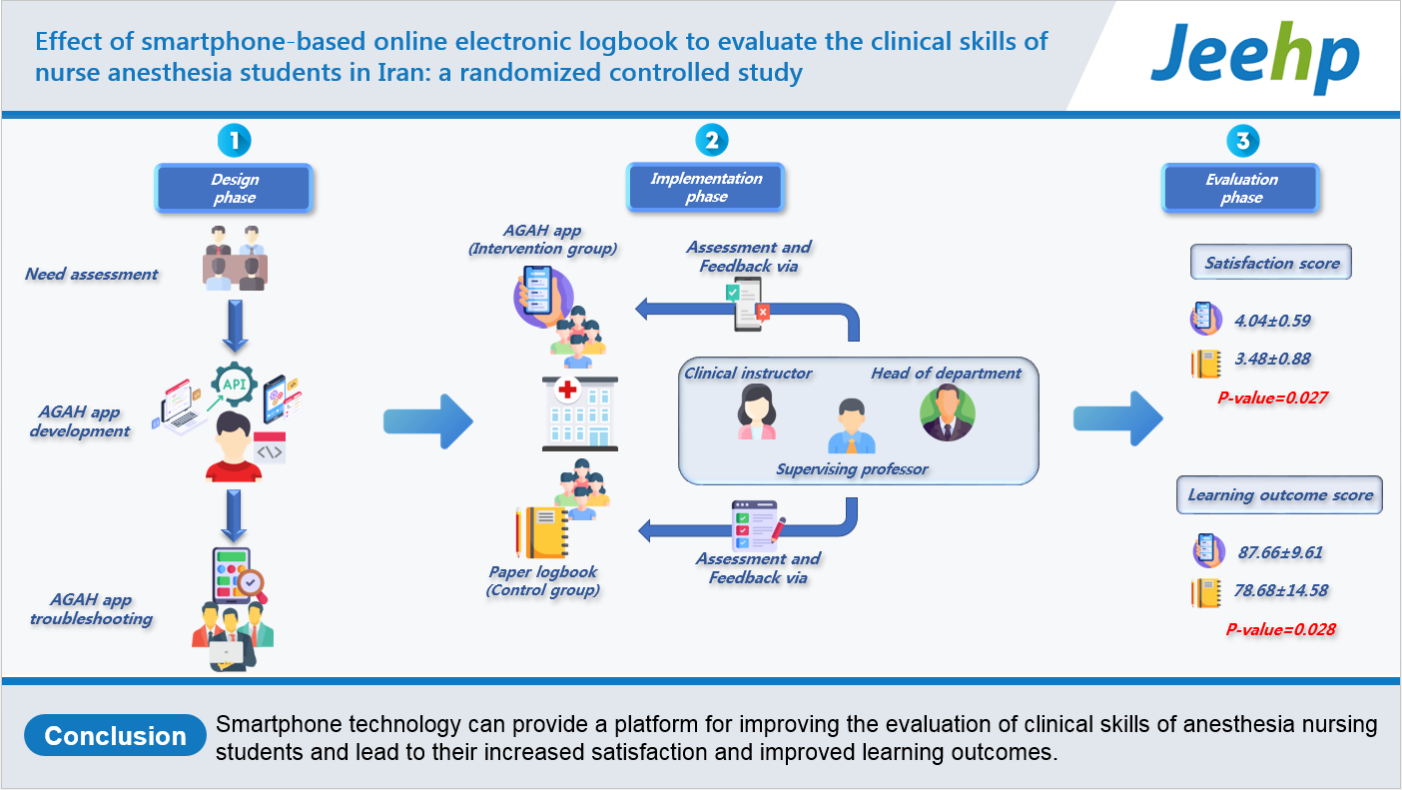


**Table 1.** Demographic characteristics of the participants (n=39)

Characteristic	Total (n=39)	Groups		P-value
		Intervention (n=20)	Control (n=19)	
Personal characteristics				
Sex				0.55^a)^
Male	12 (30.76)	7 (35.00)	5 (26.31)	
Female	27 (69.23)	13 (65.00)	14 (73.68)	
Age (yr)	21.53±3.65	22.40±4.93	20.63±0.89	0.13^b)^
Academic characteristics				
Year of study				0.42^a)^
Second year	20 (51.28)	9 (45.00)	11 (57.89)	
Third year	19 (48.71)	11 (55.00)	8 (42.10)	
Grade point average	16.80±1.36	16.73±1.44	16.88±1.30	0.72^b)^

Values are presented as number (%) or mean±standard deviation.

a) P-value obtained with the chi-square test.

b) P-value obtained with the independent t-test.

**Table 2.** Comparison of satisfaction and learning outcome between the intervention and control groups (n=39)

Variable	Group		t-value	P-value
	Intervention (n=20)	Control (n=19)		
Satisfaction	4.04±0.59	3.48±0.88	2.29	0.027^a)^
Learning outcome	87.66±9.61	78.68±14.58	2.28	0.028^a)^

Values are presented as mean±standard deviation, unless otherwise stated.

a) P-value obtained with the independent t-test.

## Footnotes

**Data availability**
Data files are available from the Harvard Dataverse: https://doi.org/10.7910/DVN/MS3UHT Dataset 1. Raw response data from participants for the satisfaction survey.

Dataset 2. Raw rating data from raters for the students’ learning outcomes.

## Supplementary materials

Supplementary files are available from the Harvard Dataverse: https://doi.org/10.7910/DVN/MS3UHT

Supplement 1. Design phase.

Supplement 2. Satisfaction questionnaire.

Supplement 3. Example of anesthesia skills evaluation checklist in the paper logbook.

Supplement 4. Audio recording of the abstract.
