# Supplementary material for: Effect of a smartphone-based online electronic logbook to evaluate the clinical skills of nurse anesthesia students in Iran: a randomized controlled study
Source: J Educ Eval Health Prof. 2023 Mar 31;20:10. doi: 10.3352/jeehp.2023.20.10 (PMC10169697; doi:10.3352/jeehp.2023.20.10)
Supplement: Supplementary file 3 — Supplement 1. Design phase. [file jeehp-20-10-suppl1.docx]

**The process of designing AGAH app**

**1) Design phase**

**a) Needs assessment**

The process of designing and developing AGAH app was modeled after the logbook approved by the Anesthesiology Department of AJUMS. First, an educational council meeting was held with the presence of the head of the department and 5 faculty members of Anesthesiology and Medical Education. After discussions and careful investigations, they admitted the need for a smartphone-based online electronic logbook as an educational tool and approved it. It should be noted that the logbook of the Anesthesiology Department has been used for several years as a valid and reliable tool to evaluate the clinical skills of nurse anesthesia students. It encompasses the completion guide, educational objectives, and a checklist for evaluation of technical and non-technical skills of anesthesia, clinical conferences, and any extracurricular activities. The skill evaluation checklist also included the number of times each procedure has to be done based on the curriculum and the student’s performance level (needs to be repeated, average, good, excellent) based on the clinical instructor’s feedback.

**b) Design and development of AGAH app**

All the necessary information and features needed to develop AGAH app were collected by reviewing the literature and other electronic logbooks designed around the world. Then, several meetings were held with the presence of our research team and the software engineering team. In these meetings, the basic framework, appearance, and the expected affordances of the application were determined with an emphasis on its online feature. Also, during a 2-hour meeting, the opinions of 4 faculty members of the Anesthesiology Department on the initial framework of the application design were reviewed and applied. Then, the engineering team started the programming and design process of AGAH app according to the mentioned framework. During this period, various software modules were gradually designed and tested by the engineering team. They were also examined by the research team so that feedback could be given to the engineering team in case there were any defects or errors, or if the appearance and performance of the application did not satisfy the research team. In addition, the features of AGAH app web management panel, database, information storage process, and application programming interfaces (APIs) were specified in advance by the engineering team and in consultation with researchers. AGAH online service includes a server that provides services to users connected to the server through RESTful API (Representational state transfer) using PHP language (Hypertext Preprocessor) and Laravel framework. It also has a management panel where user information is managed by the administrator. In order to use this service easily, the Android application is designed based on cutting edge technology and the MVVM model (Model View View Model), which performs authentication operations using token-based authentication method. Using suitable forms, it establishes connections between users and the server in order to register requests. At the end, AGAH app ver. 1.0.0 was developed as an Android-compatible application. For this purpose, the researchers and 2 faculty members of the Anesthesiology Department installed and run the AGAH app on their smart phones so that their final comments could be applied, and any possible problems could be resolved. At this stage, a number of defects in the application and its management panel were identified, and the engineering team was notified to fix them. At the end, AGAH app ver. 1.0.1 was ready for implementation. Overall, the process of designing, preparing and troubleshooting of AGAH app took about 8 months.

**c) Specifications and affordances of AGAH app**

This application granted access to four types of users, namely student, clinical instructor, supervising professor, and head of department. All of these users could log into their profile by entering their username and password (Fig. 1). AGAH app makes possible the real-time monitoring of internship and provision of online feedback by the clinical instructor, the supervising professor, and the head of department. This is the most important feature of AGAH app. It should be noted that restrictions were imposed on the access of all users, except the head of department who had access to all information and activities recorded by users in order to fully monitor the internship and observe the students, clinical instructors, and supervising professors simultaneously. Also, the web-based management panel of AGAH app was under constant management and supervision of researchers. Through this panel, the administrator could define users and special internships for each academic semester, and categorize particular anesthesia skills (Figs. 2, 3). It was also possible to remove and add any of the mentioned items at any time desired. This panel was in complete connection with the application and the content of AGAH app was controlled by this panel. In general, AGAH app made it possible for the student to choose the desired skill online during the internship hours and complete its information. This information included the name of the patient and hospital, as well as the type, time, and date of surgery (Fig. 4). As soon as the student recorded the skills, the information would appear in the user account of the clinical instructor, the supervising professor, and the head of department on their mobile phones. At the same time, the clinical instructor could give their feedback based on a Likert scale (from needs to be repeated to excellent) (Fig. 5), and the supervising professor and the head of department would approve or disapprove the procedures. Also, if necessary, they could also send feedback to the student in text format. In addition, AGAH app allowed all users to change passwords, and add notes and profile photos (Fig. 6). Since various types of information were uploaded to the system by different users, maximum security protocols were put in place.


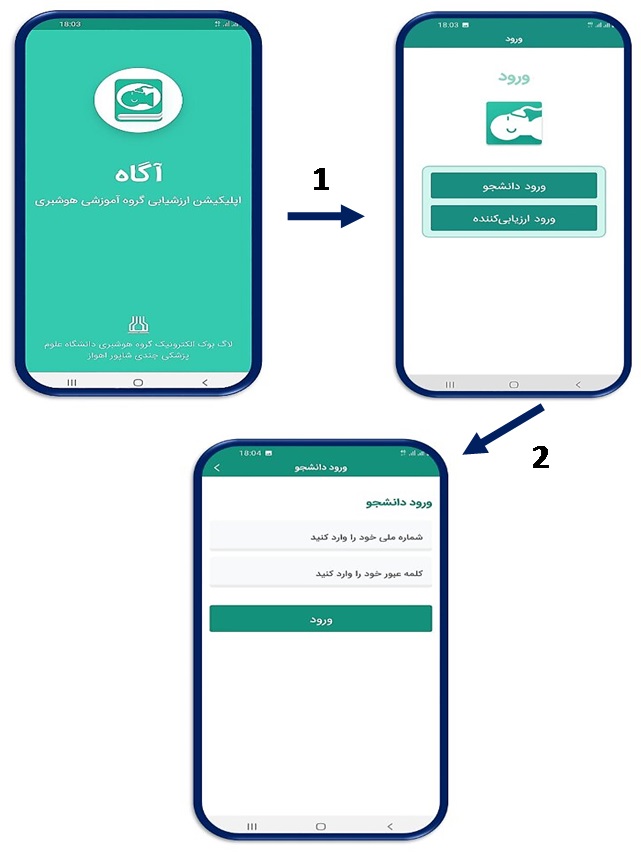


**Fig. 1.** User login involving entering username and password.


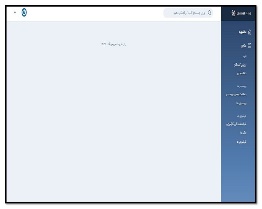


**Fig. 2.** Web-based management panel of the AGAH app.


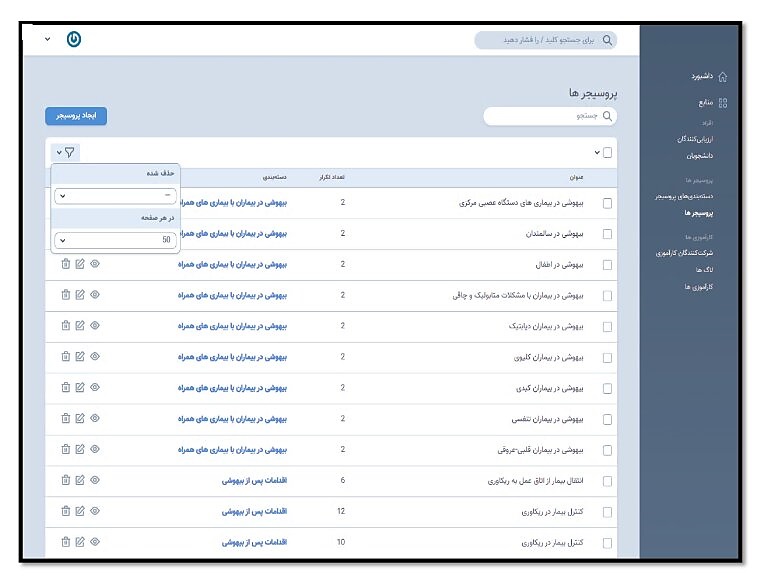


**Fig. 3.** Defining anesthesia skills in the web-based management panel of the AGAH app.


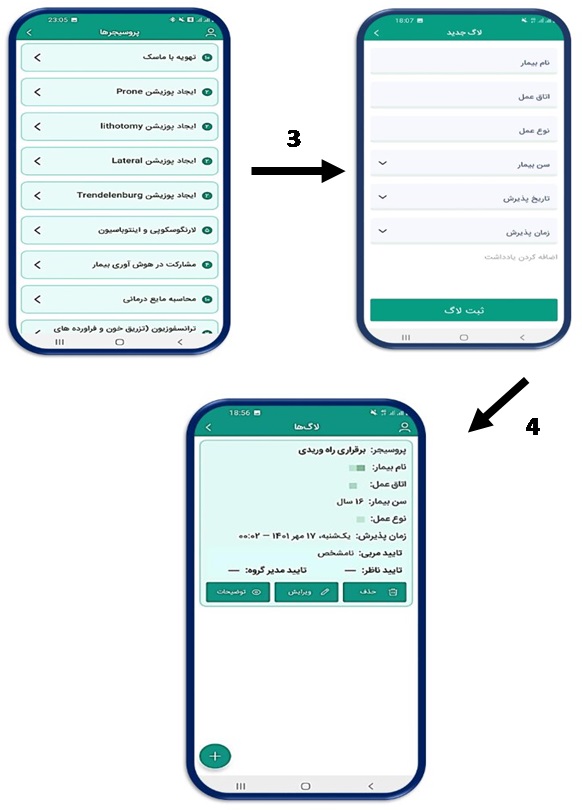


**Fig. 4.** Process of recording skills by students in the AGAH app.


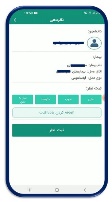


**Fig. 5.** Clinical skill evaluation by clinical instructor in the AGAH app.


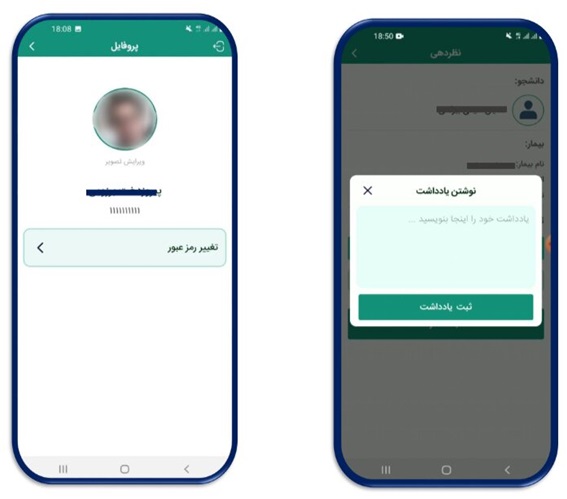


**A B**

**Fig. 6.** (A, B) Options to change password, add notes, and add profile photo.
