# Supplementary material for: Effect of a smartphone-based online electronic logbook to evaluate the clinical skills of nurse anesthesia students in Iran: a randomized controlled study
Source: J Educ Eval Health Prof. 2023 Mar 31;20:10. doi: 10.3352/jeehp.2023.20.10 (PMC10169697; doi:10.3352/jeehp.2023.20.10)
Supplement: Supplementary file 4 — Supplement 2. Satisfaction questionnaire. [file jeehp-20-10-suppl2.docx]

**Questionnaire to evaluate nurse anesthesia students’ level of satisfaction with smartphone-based online electronic/paper-based logbook evaluation**

1) Gender: 2) Age: 3) Academic year: 4) GPA:

| No. | Items | Completely agree | Agree | Have no opinion | Disagree | Completely disagree |
| --- | --- | --- | --- | --- | --- | --- |
| 1 | The guide on how to use and fill out the logbook was complete and clear. |  |  |  |  |  |
| 2 | The overall appearance of the logbook was good. |  |  |  |  |  |
| 3 | The design of the logbook pages was of good quality. |  |  |  |  |  |
| 4 | The coloring of the logbook pages was appropriate and adequate. |  |  |  |  |  |
| 5 | Working with this type of logbook was easy and comfortable for me. |  |  |  |  |  |
| 6 | The contents and items of the logbook I used were legible and clear. |  |  |  |  |  |
| 7 | I could easily record my information and activities in it. |  |  |  |  |  |
| 8 | The steps I had to go through in order to record my activities in the logbook had a logical number and sequence. |  |  |  |  |  |
| 9 | I could have access to the activities that I had previously recorded in my logbook at a very fast speed. |  |  |  |  |  |
| 10 | There was no limit to the number of procedures I could record in the logbook. |  |  |  |  |  |
| 11 | I could easily use my logbook anywhere and anytime. |  |  |  |  |  |
| 12 | By using this type of logbook, I received quick and timely feedback from my instructor or supervisor. |  |  |  |  |  |
| 13 | This type of logbook has boosted my motivation to learn more. |  |  |  |  |  |
| 14 | This type of logbook has increased my activities in clinical settings. |  |  |  |  |  |

| No. | Items | Completely agree | Agree | Have no opinion | Disagree | Completely disagree |
| --- | --- | --- | --- | --- | --- | --- |
| 15 | Using this type of logbook has accelerated the correction of my clinical activities. |  |  |  |  |  |
| 16 | Overall, this type of logbook has improved my performance in clinical activities. |  |  |  |  |  |
| 17 | Evaluation through this type of logbook has raised my self-confidence. |  |  |  |  |  |
| 18 | Using this type of logbook has improved my interaction and academic communication with my clinical instructor. |  |  |  |  |  |
| 19 | The speed of evaluation thanks to this type of logbook is very suitable. |  |  |  |  |  |
| 20 | Overall, the use of this type of logbook is very useful and suitable for evaluating clinical skills. |  |  |  |  |  |
