# Supplementary material for: Effect of a smartphone-based online electronic logbook to evaluate the clinical skills of nurse anesthesia students in Iran: a randomized controlled study
Source: J Educ Eval Health Prof. 2023 Mar 31;20:10. doi: 10.3352/jeehp.2023.20.10 (PMC10169697; doi:10.3352/jeehp.2023.20.10)
Supplement: Supplementary file 5 — Supplement 3. Example of anesthesia skills evaluation checklist in the paper logbook. [file jeehp-20-10-suppl3.docx]

**Department of Anesthesiology School of Allied Medical Sciences Internship logbook**

**of AJUMS**

| **Admission of patient in the operating room (full report of the patient’s condition including: tests, consultations, underlying diseases, etc.)** | | | | | | | | | | |
| --- | --- | --- | --- | --- | --- | --- | --- | --- | --- | --- |
| Name and surname of the patient | Date | Type of the surgery | Patient’s age | Evaluation criteria | | | | Seal and signature of the clinical instructor | Feedback of the supervising professor | Feedback of the head of department |
|  |  |  |  | Excellent | Good | Average | Needs to be repeated |  |  |  |
|  |  |  |  |  |  |  |  |  |  |  |
|  |  |  |  |  |  |  |  |  |  |  |
|  |  |  |  |  |  |  |  |  |  |  |
|  |  |  |  |  |  |  |  |  |  |  |
|  |  |  |  |  |  |  |  |  |  |  |
|  |  |  |  |  |  |  |  |  |  |  |
|  |  |  |  |  |  |  |  |  |  |  |
|  |  |  |  |  |  |  |  |  |  |  |
|  |  |  |  |  |  |  |  |  |  |  |
|  |  |  |  |  |  |  |  |  |  |  |
|  |  |  |  |  |  |  |  |  |  |  |
|  |  |  |  |  |  |  |  |  |  |  |

**“An example of anesthesia skills evaluation checklist”**
